# Pratique de l’analgésie péridurale auprès de 20 parturientes au Centre Hospitalier Universitaire Sylvanus Olympio de Lomé (Togo)

**DOI:** 10.11604/pamj.2017.26.56.11520

**Published:** 2017-02-01

**Authors:** Pilakimwé Egbohou, Tabana Mouzou, Hamza Doles Sama, Pikabalo Tchétike, Sarakawabalo Assénouwé, Gnimdou Akala-Yoba, Kadjika Tomta

**Affiliations:** 1 Service d’Anesthésie Réanimation du CHU Sylvanus Olympio de Lomé, Togo

**Keywords:** Analgésie péridurale, obstétrique, CHU, Togo, Epidural analgesia, obstetrics, University Hospital, Togo

## Abstract

Etude prospective et descriptive sur la pratique de l’analgésie péridurale (APD) obstétricale au CHU Sylvanus Olympio (CHU SO) de Lomé. Etude menée de février à juin 2014. Après accord des gestantes choisies au hasard et en l’absence de contre-indication à l’issue de la consultation d’anesthésie, faite au 8ème mois de la grossesse, des femmes ont été retenues pour l’étude. Sur 29 gestantes retenues, 20 (69%) ont bénéficiées de l’APD. Age moyen 30,6±6,6 ans, primigestes : 35%, multipares 50%, Obèses (BMI>30): 25%. Nombre moyen de ponctions: 1,2±0,5; reflux de sang dans le cathéter: 5%, brèche dure-mérienne : 0. Délai moyen d’installation: 8,5 ±2,2mn. Quantité moyenne de bupivacaine isobare à 0,125%: 28,8±8ml; Echelle Numérique à T10min < 3 pour toutes les parturientes. Bloc moteur: 0. Hypotension: 1cas (5%). Mode d’accouchement: voie basse: 19 (95%), césarienne: 1 (5%). Détresse respiratoire à la naissance du nouveau né: 0. Note de satisfaction: 9,8±0,5 /10. L’APD obstétricale est possible au CHU Sylvanus Olympio de Lomé. En attendant sa vulgarisation à toutes les parturientes par la disponibilité des moyens humains et matériels, la réaliser pour ses indications médicales serait un premier pas.

## Abstract (en)

We conducted a prospective and descriptive study on epidural analgesia (EA) at the Sylvanus Olympio University Hospital Center in Lomé from February to June 2014. After taking consent from pregnant women selected by simple random sampling and in the absence of contraindication for anesthesia (pre-anesthetic assessment performed in their 8th month of pregnancy), women were enrolled. Out of 29 selected women, 20 (69%) underwent EA. The average age was 30.6 ± 6.6 years: primiparas 35%, multiparas 50%, Obese women (BMI> 30)25%. Average number of punctures: 1.2 ± 0.5; blood reflux into the catheter: 5%; dural puncture: 0. Delay for anesthesia induction: 8.5 ± 2, 2mn. Mean dosage of 0.125% isobaric bupivacaine: 28.8 ± 8ml; digital scale at T10min <3 for all parturient women. Motor block: 0. Hypotension: 1 case (5%). Mode of delivery: vaginal births 19 (95%), cesarean section 1 (5%). Respiratory distress in the newborn: 0. Level of satisfaction: 9,8 ± 0.5 / 10. Sylvanus Olympio University Hospital Center in Lomé provides obstetric EA. While awaiting its availability to all parturient women based on human and material resources, the use of epidural analgesia in the presence of medical indications would be a first step.

## Introduction

La douleur de l’accouchement est identifiée et décrite comme l’une des plus sévères, voire insupportable [1]. L’analgésie périmédullaire (péridurale standard ou rachi péridurale combinée) pour le travail de l’accouchement est admise aujourd’hui comme la seule, réellement efficace et sûre [2]. L’acceptabilité de l’analgésie péridurale (APD) pour le travail de l’accouchement par les gestantes africaines dans leur grande majorité ne fait pas de doute [3, 4]. Sa mise en place nécessite un minimum en termes d’équipements et de personnels qualifiés qui pourrait expliquer les difficultés de son démarrage dans les maternités africaines en particulier celles du Togo. Nous avons donc voulu à travers cette étude expérimenter la faisabilité de cette APD obstétricale au Centre Hospitalier Universitaire Sylvanus Olympio (CHU SO) de Lomé au Togo.

## Méthodes

Notre étude a été prospective et descriptive sur mois 05mois, allant de février à juin 2014. Des gestantes au 8 ème mois de grossesse ont été choisies au hasard et adressées pour une consultation d’anesthésie en vue d’une APD. En l’absence de contre-indications à l’APD et après leurs accords, elles ont été retenues pour une APD dès leur entrée en travail. Le moment de pose de la péridurale était déterminé par l’obstétricien à 3 ou 4 cm de dilatation cervicale. La parturiente était préalablement mise en condition : monitorage (Pression artérielle non invasive, SPO2, scope ECG, enregistrement continu des bruits de cœur fœtaux par un tocographe), voie veineuse périphérique et remplissage par du Ringer lactate 500ml. Le matériel d’anesthésie général et de réanimation disponible et prêt à l’usage au besoin, source d’oxygène vérifiée. La ponction en position assise et la technique du mandrin liquide était celle pratiquée. Un test d’aspiration et une dose-test à la lidocaïne 2% adrénalinée à 1/200 000 sont ensuite réalisée après la pose du cathéter de péridural. Ensuite la parturiente était rallongée en décubitus dorsal et un bolus d’un mélange de bupivacaïne 0,125% : 10 ml et du fentanyl 100 mcg est injecté en doses fractionnées avec des aspirations répétées, et une surveillance des paramètres hémodynamiques. Un test du niveau sensitif au froid et d’un bloc moteur par le score de Bromage modifié était réalisé dès la 5 ème mn toute les minutes jusqu’à l’installation du bloc sensitif. L’évaluation de la douleur était faite au moyen d’une échelle numérique (EN) de 0 à 10, juste avant la pose de l’APD, à 10 min, 20 mn, 60mn, 120mn après la pose de l’APD (T0, T10, T20, T60, T120). L’entretien de l’analgésie était réalisée par des boli systématiques de bupivacaïne 0,125%, 5ml toutes les heures jusqu’à l’accouchement ou à la demande entre les boli systématique. La satisfaction des parturientes a été évaluée le lendemain de la pose de l’APD obstétricale sur une échelle numérique de 0 (pas du tout satisfaite) à 10 (entièrement satisfaite). Les données démographiques, obstétricales, anesthésiologiques ont été étudiées. La saisie et l’analyse des données ont été faites grâce au le logiciel épiinfo3.5.

## Résultats

Un total de 20 parturientes sur 29 gestantes préalablement retenues en fin de consultation préanesthésique ont bénéficié de la pose de l’APD; 4 parturientes ayant désistées au dernier moment, 3 ayant été césarisées en urgence pour souffrance fœtale aigue, et 2 autres arrivées à la maternité du CHU en phase avancée de dilatation cervicale (>8cm) et ayant accouchées presque immédiatement. L âge moyen des parturientes était de 30,6 ± 6,6 ans. Les parturientes étaient chrétiennes (80%) et musulmanes (20%). Les Primigestes représentaient 35% et les multipares 50%, parité moyenne 1,6 ±1,6. Vingt cinq pour cent des parturientes étaient obèses (BMI >30) et 55% en surpoids (BMI entre 25 et 30). La ponction était préférentiellement réalisée dans l’espace inter épineux L4 –L5 (85%) et L3-L4 (15%). L’espace péridurale a été retrouvée après une ponction dans 50% des cas, et 2 et 3 ponctions dans respectivement 35% et 15%, moyenne des ponctions : 1,2±0,5. La dose- test à la lidocaïne 2% adrénalinée à 1/200 000 a été réalisé chez toutes les parturientes et s’était révélée normale (absence de modification à l’ECG, absence de bloc moteur). Cette dose-test a été précédée de test d’aspiration négatif dans tous les cas. Aucune brèche dure-mérienne n’a été constatée lors de la ponction. Un cas (5%) de reflux de sang dans le cathéter imposant un retrait et une nouvelle ponction. Le délai moyen d’installation de l’analgésie était de 8,5 ±2,2 mn. Le niveau sensitif à 10 mn après la pose de l’APD a permis de retrouver un niveau à D12 (pubis) dans 10 %, D10 (ombilic) dans 60 % et D8 (entre ombilic et appendice xyphoïde) dans 30 % des cas. Le bloc moteur recherché par l’échelle de Brômage modifié était absent chez toutes les parturientes. La quantité moyenne de bupivacaïne 0,125% (boli cumulés) utilisée était de 28,8 ± 8 ml pour une durée moyenne du travail obstétrical de 265 ±181mn. L’évaluation de la douleur par l’EN avant la pose de l’analgésie péridurale avait permis de retrouver un score de 10 pour toutes les parturientes. Dix minutes (T10) après l’induction de l’analgésie péridurale, toutes les parturientes avaient un score < 3. Le Tableau 1 donne le détail des EN enregistrées. Comme complication immédiate un cas (5%) d’hypotension corrigée par un bolus d’éphédrine 6 mg et un remplissage vasculaire par du Ringer lactate. Au plan obstétrical, 19 (95%) parturientes ont accouchés par voie basse et une (5%) par césarienne pour une anomalie du rythme cardiaque fœtal. Aucun nouveau né n’a présenté de détresse respiratoire à l’accouchement. Aucun cas de céphalées par brèche dure-mérienne n’a été retrouvé jusqu´à 7 jours après l’APD. La satisfaction moyenne des parturientes évaluées était de 9,8±0,5 sur une échelle de 0 à 10.

**Tableau 1:** Evaluation de la douleur par l’échelle numérique (EN)

EN	T0 : n (%)	T10 : n (%)	T20 : n (%)	T60 : n (%)	T120: n (%)
0-3	0 (0)	20 (100)	20 (100)	18 (90)	12 (60)
4-6	0 (0)	0 (0)	0 (0)	2 (10)	5 (29,4)
7-10	20 (100)	0 (0)	0 (0)	0	0 (0)

T0: avant pose de la péridurale, T10: 10 minutes après pose de la péridurale, T20: 20 minutes après pose de la péridurale, T60: 60 minutes après la pose de la péridurale. T120: 120 minutes après pose de la péridurale.

## Discussion

L’APD obstétricale a été possible au CHU SO de Lomé au prix de certains aménagements en salle de travail : un box a été équipé à la maternité pour répondre au minimum de sécurité recommandé: monitorage (PANI, SPO2, ECG 3 dérivations, tocographe pour la surveillance et l’enregistrement des bruits du cœur foetaux), source d’oxygène, chariot d’urgence contenant du matériel d’anesthésie et de réanimation. La disponibilité en Kit de péridural expliquait le petit nombre de notre échantillon. Le mode d’administration dans notre étude était celui en boli par injection à travers le cathéter de péridurale par un personnel anesthésiste en raison du manque de pousse-seringues électriques pour une perfusion continue ou de pompe de PCEA (Patient Control Epidural Analgesia). Il est admis actuellement que le mode d’administration par PCEA ou plus récemment par PIEB (Programmed Intermittent Epidural Bolus) soient les meilleurs car offrant une meilleure analgésie aux parturientes tout en diminuant la consommation d’anesthésique local et donc des effets secondaires et en réduisant le nombre d’intervention du personnel anesthésique une fois l’ APD mise en route [5, 6]. La bupivacaïne a été l’anesthésique local utilisé dans cette étude, elle a été utilisée à de faible concentration 0,125% pour réduire au maximum le bloc moteur tout en procurant une analgésie optimale. Dans cette étude, une analgésie satisfaisante a été obtenue avec un délai relativement court (8min) après le premier bolus. Il n’y a pas eu d’installation de bloc moteur. La bupivacaïne serait un anesthésique local aussi efficace que la ropivacaïne dans cette indication [7]. L’association à un morphinique tel le fentanyl avait pour but d’optimiser l’analgésie et réduire les doses d’anesthésique local comme recommandé [8].

## Conclusion

L’APD obstétricale est possible dans une maternité d’un pays en développement comme le CHU Sylvanus Olympio de Lomé au Togo. Des contraintes telles la disponibilité en ressources humaines et matérielles freineront certainement sa vulgarisation à toutes les parturientes pour quelques années encore au Togo. En attendant, la réaliser pour ses indications médicales serait un premier pas.

### Etat des connaissances actuelles sur le sujet

- L’analgésie péri médullaire est le Gold standard dans la prise en charge de la douleur de l’accouchement. Largement rependu dans les pays du Nord, elle peine à s’installer dans les pays en développement du fait du manque de moyens humains et matériels.

### Contribution de notre étude à la connaissance

- Cette étude a permis de comprendre que la mise en route de l’analgésie péridurale obstétricale au Togo est possible si des moyens humains et matériels sont alloués.
